# Integrated neuropsychological assessment in patients undergoing neurosurgical and endovascular treatment of unruptured cerebral aneurysms: results of a prospective observational study

**DOI:** 10.1007/s00701-026-06803-9

**Published:** 2026-02-24

**Authors:** Marco Galeazzi, Rina Di Bonaventura, Fulvio Vincenzo Grilli, Martina Silvestri, Alessandro Olivi, Enrico Marchese, Alessio Albanese

**Affiliations:** 1 Institute of Neurosurgery, Università Cattolica del Sacro Cuore, 00168 Rome, Italy; 2 Current Institution: Department of Neuroscience, Fondazione Policlinico Universitario Agostino Gemelli IRCCS, 00168 Rome, Italy

**Keywords:** Clipping, Neurosurgery, Cognitive outcome, Quality of life

## Abstract

**Background:**

The treatment of unruptured cerebral aneurysms has evolved through new surgical and endovascular approaches. Its goal is to ensure the patient survival with a good quality of life, so cognitive outcome should be considered a critical factor in the decision-making analysis. This study aimed to investigate the impact of unruptured cerebral aneurysm treatment on cognitive function.

**Method:**

This prospective study enrolled 109 patients with unruptured cerebral aneurysms, 65 treated with microsurgery and 44 with endovascular procedures. Cognitive function was assessed using the Montreal Cognitive Assessment (MoCA) test, anxiety and depression using the Hospital Anxiety and Depression Scale (HADS), and quality of life by the SF-36 questionnaire. Potential risk factors influencing the neuropsychological assessment were recorded. Patients were evaluated before treatment, at discharge and at 6-months, 1-year and 2-years post treatment.

**Results:**

Before treatment, 24 patients exhibited a cognitive impairment on the MoCA test. These 24 cases were significantly older and had higher incidence of anxiety or depression under treatment. At 6 months two patients with preoperatively normal cognitive function developed a new deficit: they presented transient post-surgical complications and investigated risk factors. These two patients recovered completely at 12 months. Among those with baseline impairment 10 patients recovered at 6 months and 4 more at 12 months. At 24 months, 99 patients presented with normal MoCA scores and 10 patients showed persistent preoperative deficit.

**Conclusions:**

Neither minimally invasive microsurgery nor endovascular treatment was associated with a decline in global cognitive function within two years of follow-up. Anxiety and depression symptoms tended to improve post treatment, while quality-of-life scores remained stable. Transient postoperative complications did not influence long term cognitive outcomes. These findings highlight the importance of recognizing patient and aneurysm related risk factors that may predispose to cognitive decline. Individualized planning and pre-treatment neuropsychological assessment are therefore essential.

## Introduction

The treatment of unruptured cerebral aneurysms has undergone significant evolution over the past two decades, driven by advances in both surgical and endovascular techniques.

Milestones in modern vascular neurosurgery include the adoption of minimally invasive approaches that limit cerebrovascular dissection, the introduction of intraoperative neurophysiological monitoring and the implementation of flow-preserving techniques such as micro-Doppler sonography and Indocyanine Green video-angiography.

At the same time, endovascular procedures have continuously evolved through the development of new devices and technologies.

These improvements have led to an increased patient survival with better functional outcome and higher patient satisfaction.

In the past, the primary goals of treatment were complete aneurysm exclusion and patient survival, today it is equally essential to achieve these results while minimizing complications and morbidity, thereby ensuring a good quality of life for the patient.

It is well established that subarachnoid hemorrhage resulting from ruptured intracranial aneurysms can impair cognitive and neuropsychological functioning [3, 8, 13, 15, 18, 20, 22, 23, 31, 33].

However, the impact of unruptured cerebral aneurysms and their treatment on cognitive performance remains insufficiently studied.

Some investigations have reported that microsurgical clipping of unruptured aneurysms does not cause major cognitive dysfunctions [14, 19, 21, 24], whereas others have found that patients treated with endovascular coiling exhibit significantly fewer and less severe cognitive deficits compared to those who undergo surgical clipping [5]. There is, however, general agreement that treatment tends to improve symptoms of anxiety and depression [14, 29].

The most recent meta-analysis on the subject concluded that no definitive conclusions could be drawn due to the scarcity of studies addressing this issue, the heterogeneity of cognitive assessment tools, and the limited size of reported case series [4].

Current studies on the topic share several limitations:

- small sample sizes;
- restricted age ranges among participants;
- heterogeneous populations, with some studies focusing on specific aneurysm locations while excluding others (e.g., anterior communicating artery aneurysms, whose treatment may affect frontal lobe cognitive functions);
- inconsistent timing of neuropsychological assessments with follow-up periods ranging from one week to three months, and a lack of long-term evaluations such as those conducted in the present study;
- inclusion of both ruptured and unruptured aneurysms within the same analysis.
- Therefore, the present study provides a prospective and longitudinal evaluation of cognitive outcomes following surgical and endovascular treatment for unruptured cerebral aneurysms.

## Materials and methods

### Institutional case series

109 patients treated at our Institution for unruptured cerebral aneurysms between January 2021 and September 2023 were enrolled. Among them, 65 patients underwent microsurgical clipping, and 44 received endovascular aneurysm occlusion.

In our center, patients with cerebrovascular diseases are discussed in a multidisciplinary setting with interventional neuroradiologists at a weekly meeting. Here, the morphological characteristics of the aneurysm and the patient's clinical condition are analysed, on the basis of which the best therapeutic strategy is established collegially. The case series included in this study is not intended to be representative of the overall treatment distribution at our center during the study period, as patients who declined participation and, to a lesser extent, those who underwent endovascular treatment but were admitted to another ward were not included.

Demographic and clinical characteristics were collected for all patients, including factors that could influence neuropsychological assessment, such as the presence of psychiatric disorders under treatment with psychotropic medications, family history of cerebral aneurysm, previous subarachnoid hemorrhage, and epilepsy under treatment. No statistically significant differences were observed between the surgical and endovascular groups in these baseline characteristics (Table 1).

**Table 1:** Demographic characteristics of the population

	All patients *n* = 109	Surgical treatment *n* = 65	Endovascular treatment *n* = 44	*p* value
Mean age (mean ± SD)	58,1 ± 13,5	57,2 ± 11,9	59,5 ± 15,3	Ns
Sex (F/M)	83/26	50/15	33/11	Ns
Anxiety or depression in treatment (%)	17 (15,6%)	11 (16,9%)	6 (13,6%)	Ns
Current antiepileptic therapy (%)	5 (4,6%)	3 (4,6%)	2 (4,5%)	Ns
mRS 0–1 (%) 2–3(%)	107 (98,2%) 2 (1,8%)	65 (100%) 0 (0%)	42 (95,5%) 2 (4,5%)	Ns

Abbreviations: *F* Female, *M* Male

*mRS* modified Rankin Score, *Ns* not significant, *SD* standard deviation

Table 2 summarizes the characteristics of the treated aneurysms. Aneurysms managed surgically were significantly smaller than those treated endovascularly (median diameter 6 mm versus 7 mm, p = 0.0073).

**Table 2:** Characteristics of aneurysms

	All patients *n* = 109	Surgical treatment *n* = 65	Endovascular treatment *n* = 44	*p* value
Maximum diameter median [min, max] mm	7 mm [2;37]	6 mm [2;25]	10 mm [3; 37]	0,0073
Multiple (%)	23 (21,1%)	13 (20%)	10 (22,7%)	Ns
Location:				Ns
ACM	44 (40,4%)	44 (67,7%)	0 (0%)	Ns
ICA	24 (22%)	3 (4,6%)	21 (47,7%)	Ns
ACom	26 (23,9%)	13 (20%)	13 (29,5%)	Ns
Other	15 (13,8%)	5 (7,7%)	10 (22,7%)	Ns
Post-surgery antiepileptic therapy	2 (1,8%)	1 (1,5%)	1 (2,3%)	Ns
Neurological complications	4 (5,5%)	2 (3%)	2 (4,5%)	Ns

Abbreviations: *ACM* middle cerebral artery, *ICA* internal carotid artery, *ACom* anterior communicating artery, *Max* maximum, *Min* minimum

Details of the surgical approaches and endovascular devices used are presented in Table 3***.***

**Table 3:** Treatment characteristics

Surgical Approaches	n (%)	Endovascular Devices	n(%)
MPT (%)	40 (61,5%)	Flow diverter (%)	26 (59,1%)
LSO (%)	15 (23,1%)	Coil + stenting	14 (31,8%)
PT standard: Parasagittal Suboccipital	7 (10,8%) 2 (3,1%) 1 (1,5%)	Coil	4 (9,1%)

Abbreviations: *LSO* lateral supraorbital, *MPT* mini-pterional, *PT* pterional

The standard Pterional approach was employed in 7 cases, including middle cerebral artery (MCA) aneurysms larger than 10 mm, one patient with both Anterior Communicating artery (ACom) and MCA aneurysms and one case of a 12 mm internal carotid artery (ICA) ophthalmic segment aneurysm.

Among endovascular procedures, flow diverter stents were used in 59.1% of cases, reflecting the predominance of ICA aneurysms in this subgroup. In 31.8% of cases, a combination of stent-assisted coiling was performed, mainly for ACom and Basilar artery aneurysms, while 9.1% of patients were treated with coiling alone.

### Study protocol

All patients underwent a comprehensive and standardized neuropsychological assessment using a uniform set of tests selected in collaboration with a neuropsychologist and administered by the same investigator.

The clinical condition at admission was evaluated using the Modified Rankin Scale (mRS) [25].

Cognitive function was assessed with the validated Italian version of the Montreal Cognitive Assessment (MoCA), which explores eight cognitive domains: verbal short-term and delayed memory, visuospatial skills, executive functions, attention, concentration, working memory, and orientation in time and space [6, 27].

The test includes various visuospatial tasks and procedures specifically designed to assess frontal/executive functions, attention and long-term memory [17, 28].

According to standardized statistical procedures for neuropsychological testing in the Italian population, normalized cut-off scores were calculated for age and education for each cognitive domain, and raw scores were converted into equivalent standardized scores [6].

The Hospital Anxiety and Depression Scale (HADS) was administered to detect the presence of anxiety and depressive symptoms [9].

In this study, the test was not intended to diagnose clinical anxiety or depression but rather to provide a quantitative measure of emotional distress to aid the interpretation of cognitive performance (MoCA results).

HADS consists of 14 items: 7 assessing anxiety (HADS-A) and 7 assessing depression (HADS-D). Each question is rated on a four-point scale (0 = not at all to 3 = definitely yes), yielding a subscale score ranging from 0 to 21 for both anxiety and depression, and a completed total score (HADS-T) representing overall emotional symptomatology. Patients answered based on their emotional state over the previous week.

Health-related quality of life (QoL) was assessed using the Medical Outcomes Study 36-Item Short Form Health Survey (SF-36), a validated and reliable measure of quality of life in vascular disease [2, 32].

The Italian version of the SF-36, which demonstrates good sensitivity and specificity [1], consists of 36 items divided into 8 domains: physical functioning, role limitations due to physical health, role limitations due to emotional problems, energy/fatigue, emotional well-being, social functioning, pain and general health perception.

These domains were specifically interpretated by an expert neuropsychologist to merge these information with those deriving from the HADS scale in order to relate the changes in the anxiety/depression scores to the pathology and its treatment.

The study protocol comprised three stages:

1. *clinical and neuropsychological pre-treatment evaluation*: two days before treatment, patients underwent neuropsychological assessment with the MoCA, HADS and SF36 test battery. Clinical history was collected including psychiatric disorders under treatment, epilepsy, and any previous subarachnoid hemorrhage related to another aneurysm in the case of multiple aneurysms. Demographic data (sex, age, education level) were recorded, and the patient’s functional status was rated according to the Modified Rankin Scale (mRS).
2. *Post treatment evaluation at discharge:* at discharge, the clinical outcome was assessed using the Glasgow Outcome Scale (GOS) [16]. A final neurological examination was performed to identify any treatment-related complications.
3. *Follow-up evaluations***:** at 6 months, 1 year and 2 years after treatment, patients underwent clinical, radiological and neuropsychological follow-up assessments. The same neuropsychological test battery (MoCA, HADS, SF-36) was administered at each follow-up.

The results of the scales, normalized for age and education, were compared between pre- and post-treatment assessments. Longitudinal analysis was conducted to evaluate changes over time and to identify any treatment-related acceleration in cognitive decline.

### Data collection, statistical analysis and human ethics

All patients provided written informed consent, and it was explicitly explained that the interview included psychological and cognitive assessments, specifically the MoCA, HADS and SF-36 tests.

Statistical analyses were performed with GraphPad Prism version 10.1.0 (GraphPad Software, La Jolla, California, USA, 2023). Descriptive statistics were reported as mean ± standard deviation (SD) and range for continuous variables, and as frequency and percentage for categorical variables. In univariate analysis, the unpaired Student’s *t*-test was used to compare means of continuous variables and the two-sided Fisher’s exact test was applied to compare categorical variables.

A p-value < 0.05 was considered statistically significant.

### Human ethics and informed consent

Due to the observational nature of the study and being all the procedures part of the routine care, no ethical approval was required. The study was conducted in accordance with the ethical standards of the institutional and national research committee and with the 1964 Helsinki Declaration and its later amendments or comparable ethical standards.

Informed consent to participate was obtained from all individual participants included in the study.

## Results

### Baseline (Time 0—Before Treatment)

Analysis of age- and education-adjusted MoCA scores before treatment identified two subgroups of patients:

- 85 patients (78%) without cognitive deficits, and
- 24 patients (22%) with pre-treatment cognitive impairment.

Patients with baseline cognitive impairment were significantly older (*p* < 0.01) and had a higher incidence of anxiety or depressive symptoms (*p* < 0.05). There were no significant differences in sex distribution, treatment modality or aneurysm location between groups (Table 4).

**Table 4:** Demographic, anamnestic, and clinical features at time 0

	Cognitive Impairment (24)	No Cognitive Impairment (85)	*P* value
Median age	65,5	57	0,0009
Anxiety or depression in treatment(%)	7 (29,2%)	14 (16,5%)	0,05
Current antiepileptic therapy (%)	2 (8,3%)	3 (3,5%)	0,068
HADS > 16	7 (29,2%)	40 (47%)	NS

Abbreviations: *HADS* Hospital Anxiety and Depression Scale, *NS* not significant

### Clinical evaluation at discharge

At discharge, the Glasgow Outcome Scale (GOS) score was 5 in 90.2% of patients and 4 in 9.8%. Among those with GOS score of 4, six were in the endovascular group (13%) and six in the surgical group (7.7%).

Postoperative complications occurred in 4 patients (5.5%), evenly distributed between the two treatment groups (two surgical and two endovascular). All cases involved symptomatic ischemic events. No procedure-related mortality was observed.

### 6 Months follow-up (Time 1)

At 6 months, among the 85 patients who had normal pre-treatment MoCA scores, 83 (97.6%) maintained normal cognitive performance. Two new transient cognitive deficits were observed: one in the surgical group and one in the endovascular group (Table 5).

**Table 5:** 6 months after treatment: Characteristics of the group without previous cognitive impairment

	No cognitive impairment before treatment (85)	No Cognitive impairment after treatment: 83, (97%)	Cognitive impairment after treatment: 2 (3%)	P value
Median age	57	56,5	74,5	0,0013
Anxiety or depression in treatment	15 (15,8%)	14 (16,8%)	0 (0%)	NS
Antiepileptic therapy	7 (8,2%)	6 (7,2%)	1 (50%)	0,0225

Abbreviations: *NS* not significant

These patients recorded a decrease in MOCA scores of respectively 3 and 6 points and the most affected domains were visuospatial and memory.

Among the 24 patients with pre-treatment cognitive impairment, 10 (41.7%) demonstrated recovery of cognitive function at this stage.

These patients recorded an increase in MOCA scores, with average increases of 4.3 points (up to maximum increases of 8 points); the domains that increased the most were memory, visuospatial and attention, with the remaining domains remaining stable.

Improvement was more frequent among younger patients and those with lower baseline anxiety/depression scores, although the difference did not reach statistical significance (p = 0.08). The distribution of demographic and clinical factors across subgroups is shown in Table 6*.*

**Table 6:** 6 months after treatment: features of the group with previous cognitive impairment

	Cognitive impairment before treatment (24)	Persistent impairment (14)	Recovered (10)	P value
Median age	64,2	72	63	0,05
Anxiety or depression in treatment	7 (29%)	5 (35%)	2 (20%)	NS
Antiepileptic therapy	2 (8,3%)	1 (7,1%)	1 (10%)	NS
HADS > 16	9 (37,5%)	6 (42%)	3 (30%)	NS

Abbreviations: *HADS* Hospital Anxiety and Depression Scale, *NS* not significant

### 12 months Follow- up (Time 2)

At 12 months, the following subgroups were identified:

- 83 patients without pre-treatment deficits maintained normal MoCA scores;
- 2 patients with new deficits at 6 months showed complete recovery. These two patients showed an increase of 4 and 3 points respectively in the MoCA score, with improvements mainly in the visuospatial and language domains, while the remaining domains remained stable.
- 10 patients had persistent preoperative cognitive deficits; 4 patients improved from impaired scores at 6 months to normal at 12 months; These patients showed an average increase of 3,5 points, with improvements mainly in the visuospatial and attention domains.

4 patients improved from impaired scores at 6 months to normal at 12 months; These patients showed an average increase of 3,5 points, with improvements mainly in the visuospatial and attention domains.

10 patients with preoperative deficits showed stable normal results at both 6- and 12- month follow-ups.

### 24 months follow up (Time 3)

At 24 months, cognitive outcomes remained stable: 99 patients (90.8%) had normal MoCA scores, and 10 patients (9.2%) continued to show persistent pre-treatment deficits. No new cognitive impairments were recorded after the first year. Anxiety and depression scores (HADS) showed a trend toward improvement in both treatment groups, while quality-of-life scores (SF-36) remained stable across all follow-up periods.

At the final evaluation, 7 patients (6,4%) presented an incomplete occlusion of the aneurysm imaging follow up. 2 endovascular patients (4,5%) presented a residual neck (Roy-Raymond class 2 [26]) while 5 patients from the surgical group (7,6%) presented in 4 cases a neck residual (Roy-Raymond class 2) left intentionally to preserve the outflow of an emerging branch, and one was an aneurysm residual (Roy-Raymond class 3) of a giant ACM aneurysm.

## Discussion

Cognitive outcome after treatment should be considered a critical factor in the decision-making process for managing unruptured cerebral aneurysms. Achieving both optimal physical and cognitive outcomes must be regarded as the ultimate goal when choosing the most appropriate treatment strategy. The surgical approach employed in treating an unruptured aneurysm can significantly influence postoperative cognitive function.

In the 1990 s and early 2000 s, authors such as Hütter et al. [10–12] and Tuffiash et al. [30] observed that microsurgical clipping via standard pterional or frontotemporal craniotomies could result in frontal and memory deficits, particularly in cases involving anterior communicating artery (ACom) or middle cerebral artery (MCA) aneurysms. These impairments were attributed to mechanical retraction of the frontal lobe, ischemic injury to perforating arteries, and subtle damage to basal forebrain structures. Since the frontal lobe plays a central role in higher cognitive functions and working memory, approaches involving minimal brain manipulation -such as the minipterional (MPT) approach- tend to have fewer cognitive repercussions compared with more invasive approaches, including the latero-supraorbital (LSO) and standard pterional (PT) approaches [7].

With the evolution of endovascular techniques, which eliminate the need for direct brain manipulation, questions have been raised regarding whether, despite surgical advances, open treatment may still accelerate cerebral aging processes or contribute to cognitive deficits that impact quality of life.

This prospective, longitudinal study aimed to address that issue by evaluating cognitive, emotional, and quality of life outcomes in patients treated for unruptured cerebral aneurysms. The goal was to promote a patient-centered approach that incorporates pre-treatment neuropsychological assessment into treatment planning thereby identifying individuals at greater risk of postoperative cognitive decline and optimizing the choice between microsurgical and endovascular treatment.

A total of 109 patients were enrolled −65 treated microsurgically and 44 endovascularly – and were evaluated using standardized battery consisting of the MoCA, HADS and SF-36 at baseline, discharge, and at 6 months, 1 year, and 2 years after treatment.

*At time 0 (before treatment)* 24 (22%) demonstrated cognitive impairment. Pre-treatment deficits correlated significantly with older age (p = 0.0009) and presence of psychiatric disorders under treatment (p = 0.05). No significant differences were observed in sex, aneurysm characteristics, or treatment modality. Notably, although anxiety and depression scores (HADS) were generally higher among patients without cognitive deficits, elevated emotional distress was observed in both groups.

*At 6 months* 83 of the 85 patients (97.6%) who had normal baseline MoCA scores maintained normal cognitive performance. Among these 65.5% exhibited a decrease in HADS scores, indicating an improvement in anxiety and depressive symptoms, with parallel stability or improvement in SF-36 scores across all patients.

Two patients developed new cognitive deficits – one in the endovascular group and one in the surgical group.

The endovascular case involved an 80 year old patient treated with a flow-diverter stent for a giant, partially thrombosed left ICA aneurysm, with no psychiatric or epileptic history.

The surgical case involved a 69 years old woman treated for an MCA aneurysm via a classical Pterional approach, complicated by a fronto-insular ischemic event with transient paresis of the left upper limb. She was also on antiepileptic therapy for seizures that occurred several weeks before surgery.

Among the 24 patients with pre-treatment impairment, 10 (41.7%) demonstrated cognitive recovery at 6 months. Persistent impairment was associated with higher age and greater emotional distress, whereas recovery correlated with improvements in both HADS and SF-36 scores.

When comparing patients with initial impairment, those who recovered at 6 months were generally younger, more frequently underwent the MPT approach and had a lower prevalence of psychiatric disorders or psychotropic medication use.

*At time 2 (12 months after treatment),* the two patients with new deficits documented at 6 months after treatment fully recovered.

**Fig. 1:**
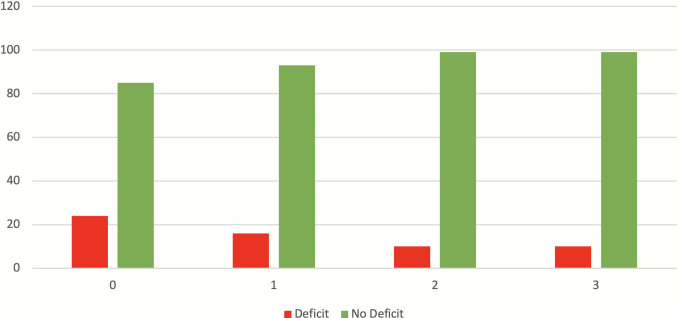
Trend of cognitive deficits at different follow-up times. Time 0: pre-treatment assessment*.* Time 1: 6 months after treatment*.* Time 2: 12 months after treatment*.* Time 3: 24 months after treatment

10 patients (8.1%; 5 surgical and 5 endovascular) had persistent cognitive impairment at all time points. At 24 months, no new cognitive deficits was recorded. Minor variations in HADS scores likely reflected personal life circumstances rather than treatment effects. Persistent impair at cognitive testing was associated with advanced age, psychiatric comorbidities and natural progression of cognitive aging (Fig. 1).

All the patients with an incomplete occlusion at imaging follow-up belonged to the subgroup that presented with a normal cognitive function throughout all the study phases. Instead, all patients with an impaired cognitive function had a complete occlusion of the aneurysm (Roy-Raymond class 1).

Being these residuals stable on seriate imaging and since the patients had been informed about the reduction in rupture risk achieved through treatment, it is plausible that their presence did not adversely affect cognitive function or the level of disease-related concern.

The findings of this study indicate that neither microsurgical nor endovascular treatment for unruptured cerebral aneurysms is associated with long-term cognitive deterioration.

On the contrary, cognitive function often improved in patients with pre-existing impairment, particularly when emotional distress is alleviated after treatment.

Transient postoperative complications such as ischemic events did not lead to sustained cognitive decline.

Rather psychological factors (subclinical symptoms of anxiety or depression), advanced age and pre-existing psychiatric or epileptic conditions emerged as the most relevant predictors of poor cognitive outcome.

The integration of pre-treatment neuropsychological assessment is therefore essential to identify at-risk patients and to personalize treatment planning.

Minimally invasive surgical approaches such as MPT should be preferred when feasible as they are associated with reduced cortical manipulation and better cognitive preservation.

## Limitations

This study has several limitations. First, it represents a preliminary, single-center experience and does not include a control group of healthy subjects, which would have allowed for a more robust comparison of cognitive trajectories over time. Second, there is heterogeneity within the study sample, particularly regarding aneurysm location and patient age, which may introduce variability in neuropsychological outcomes. Third, the study did not systematically assess medical comorbidities that could influence quality of life and emotional state. This is particularly relevant since a subset of patients had oncological comorbidities with aneurysms discovered incidentally. Such conditions may have affected HADS and SF-36 results independently of aneurysms treatment. Although the cohort size was relatively large and the follow-up period extended to 24 months, multicenter validation would strengthen the generalizability of these findings. Additionally, while standardized neuropsychological instruments were employed, incorporating more detailed, domain-specific cognitive tests in future studies could further elucidate subtle cognitive changes over time.

## Conclusions

In the past era, when cerebral aneurysm treatment was exclusively surgical, the primary goal was the complete exclusion of the aneurysm to prevent rupture and reduce mortality -often regardless of postoperative morbidity. With the advent of endovascular procedures and the continuous evolution of microsurgical techniques, the focus has shifted toward minimizing treatment-related complications and preserving quality of life. Surgical practice has advanced through the adoption of minimally invasive approaches, which reduce cerebrovascular dissection and frontal lobe manipulation, thereby decreasing the risk of seizures and potential acceleration of cognitive decline. Against this background, the central question of this study was whether current surgery might still accelerate cerebral aging compared with endovascular treatment. This study explored both aneurysm-related (site, approach) and patient-related (age, psychiatric comorbidities, use of antiepileptic or psychotropic drugs) risk factors that may predispose individuals to cognitive decline following treatment. These variables should be considered when determining the optimal therapeutic strategy. Our findings indicate that neither minimally invasive surgery nor endovascular treatment was associated with a deterioration in global cognitive function during a two year follow-up period. Moreover, anxiety and depression symptoms tended to improve after treatment, while quality-of-life scores remained stable. Patients who developed transient cognitive deficits at six months typically related to short-term postoperative complications -showed complete recovery by twelve months, mirroring their clinical improvement. Such transient events therefore did not appear to influence long-term cognitive outcomes. Persistent cognitive impairment occurred exclusively in patients with pre-existing deficits and identifiable risk factors. In contrast, increases in MoCA scores were consistently associated with reductions in HADS scores, reflecting an interdependence between cognitive and emotional well-being.

In contemporary practice, treatment decisions for unruptured cerebral aneurysms must be individualized. Incorporating pre-treatment neuropsychological assessment into the overall clinical evaluation represents a crucial step in tailoring management, identifying patients at risk, and ultimately ensuring the best possible functional and cognitive outcomes.

## Data Availability

The authors confirm that the data supporting the findings of this study are available within the article. Original database is available from the corresponding author, [R.D.B], upon reasonable request.

## Abbreviations

*ACom*: Anterior communicating artery
*CT*: Computerized tomography
*GOS*: Glasgow Outcome Scale
*ICA*: Internal carotid artery
*HADS*: Hospital anxiety and Depression scale
*LSO*: Lateral supraorbital
*MCA*: Middle cerebral artery
*MPT*: Mini-pterional
*MoCA*: Montreal Cognitive Assessment
*mRS*: Modified Rankin Scale
*NS*: Not significant
*PT*: pterional
*QoL*: Quality of life
*SF-36*: Short Form Health Survey 36
